# Simultaneous regulation of both lignin and cellulose biosynthesis modifies xylem fiber properties in *Populus*

**DOI:** 10.3389/fpls.2025.1646316

**Published:** 2025-08-04

**Authors:** Jian Li, Yu Han, Xianwen Lu, Xinwei Tang, Jiayan Sun, Meng Li, Laigeng Li

**Affiliations:** ^1^ Yuelushan Laboratory, College of Life Science and Technology, Central South University of Forestry and Technology, Changsha, China; ^2^ Key Laboratory of Plant Carbon Capture, Chinese Academy of Sciences (CAS) Center for Excellence in Molecular Plant Sciences, Chinese Academy of Sciences, Shanghai, China; ^3^ State Key Laboratory for Quality and Safety of Agro-Products, Institute of Virology and Biotechnology, Zhejiang Academy of Agricultural Sciences, Hangzhou, China; ^4^ Key Laboratory of Biodiversity Conservation in Southwest, State Forestry Administration, Southwest Forestry University, Kunming, China

**Keywords:** lignin, *Populus*, cellulose, cell wall, fiber cell

## Abstract

**Introduction:**

Wood is primarily made up of secondary xylem cell walls, with lignin, cellulose, and hemicellulose as the main chemical components. The presence of lignin represents recalcitrance to wood pulping and biofuel conversion. Consequently, reducing lignin content is a key approach to improving wood properties and optimizing its processing.

**Methods:**

In this study, we suppressed lignin biosynthesis by overexpressing a mutated transcription repressor *PdLTF1^AA^* and enhanced cellulose synthesis simultaneously by introducing cellulose synthase genes, *PdCesA4*, *PdCesA7A*, or *PdCesA8A*, specifically in xylem fiber cells.

**Results and discussion:**

The transgenic plants exhibited decreased lignin content and a significant increase in cellulose content. Transcriptome analysis indicated that expression of *PdLTF1^AA^* along with *PdCesA4*, *PdCesA7A*, or *PdCesA8A* in fiber cells resulted in transcriptional alterations in the genes associated with cell wall remodeling and polysaccharide synthesis during xylem development. The results also indicated that the diameter of wood fiber cells within the xylem is increased, which leads to a larger stem diameter in the transgenic plants. This study suggests that the biosynthesis of lignin and cellulose can be simultaneously modified, which presents a new strategy for modifying wood fiber characteristics for more efficient fiber and biomass processing.

## 1 Introduction

Wood, as a renewable biomass resource, is widely used in pulping and papermaking, as well as the potential for biofuel production (Luo and Li, 2022; Zhu and Li, 2024). Its basic structural components are cellulose, lignin, and hemicellulose. Lignin, among these components, represents significant barriers in biomass processing and usage due to its high chemical stability. The presence of lignin complicates the processing of biomass and significantly increases associated economic expenses (Ye and Zhong, 2015; Li et al., 2024). As a result, reducing lignin content has been a major study emphasis in the field of wood property modification and enhancement.

Lignin biosynthesis is derived from phenylpropanoid pathway (Boerjan et al., 2003; Li et al., 2024). Starting from phenylalanine, the pathway involves a series of enzymatic reactions catalyzed by key enzymes, including phenylalanine ammonia lyase (PAL), 4-coumarate: CoA ligase (4CL), cinnamoyl-CoA reductase (CCR), and cinnamyl alcohol dehydrogenase (CAD), which convert phenylalanine into various intermediates for monolignol synthesis. The activities of these enzymes directly influence the rate and efficiency of lignin synthesis, thus playing a crucial role in determining the quantity and quality of lignin synthesized (Hu et al., 1999; Li et al., 2003; Bryant et al., 2020; Cao et al., 2020; De Meester et al., 2022; Zhu and Li, 2024). Numerous studies have reported reducing lignin content in poplar trees through genetic manipulation of the monolignol biosynthesis pathway genes (Chanoca et al., 2019; Li et al., 2024; Zhu and Li, 2024). For instance, the expression of the *4CL* gene was successfully suppressed in *Populus*, resulting in a significant reduction of lignin content in transgenic trees (Hu et al., 1999; Li et al., 2003; Wang et al., 2012; Cao et al., 2020). Similarly, the inhibition of *4CL* and *C4H* gene expression via antisense RNA technology led to significant decreases in lignin content and the syringyl lignin (S-lignin) in transgenic *Populus* trees under both greenhouse and field conditions (Bjurhager et al., 2010; Voelker et al., 2010). Furthermore, the downregulation of *C3H* and *CAD* gene expression using RNA interference technology also achieved a substantial reduction in lignin content in transgenic trees (Ralph et al., 2012; Van Acker et al., 2017). Additionally, the knockout of *PAL*, *CCoAOMT1/2*, and *CCR2* genes using CRISPR-Cas9 technology has been shown to effectively reduce lignin content (De Meester et al., 2020; Sulis et al., 2023).

Direct manipulation of genes in the monolignol biosynthesis pathway leads to alterations in lignin accumulation but also disrupts phenylpropanoid metabolic flow, affecting plant growth (Du and Groover, 2010; Voelker et al., 2011).

Lignin biosynthesis is intricately regulated by a multitude of transcription factors and microRNAs. Therefore, modification of the regulatory factors may help to bypass the disruption of metabolic flow caused by directly modifying the lignin biosynthesis genes.

Overexpression of *miR408* has been shown to significantly reduce lignin content, decrease the ratio of syringyl lignin (S-lignin) to guaiacyl lignin (G-lignin) monomers, and consequently enhance the utilization efficiency of wood (Guo et al., 2023). Overexpression of *MYB115* can suppress the expression of lignin biosynthesis genes, resulting in a reduction in both the content of S-lignin monomers and the total lignin content (Fan et al., 2022). In our studies, a lignin-related transcription factor, *PdLTF1*, that modulates the entire monolignol biosynthesis pathway, is identified in *Populus* for its repressive role in lignin biosynthesis (Gui et al., 2019). Because the *PdLTF1* repression function can be released via phosphorylation, mutation of *PdLTF1* at its phosphorylation sites (PdLTF1^AA^) transforms it into a stable repressor (Gui et al., 2019). Thus, the mutant PdLTF1^AA^ can be utilized to engineer the monolignol biosynthesis pathway while not showing growth penalty (Gui et al., 2020).

Concurrently, cellulose is synthesized by cellulose synthase (CesA). It is believed that multiple CesAs aggregate into a cellulose synthase complex (CSC) localized on the plasma membrane to catalyze the synthesis of cellulose microfibrils (Paredez et al., 2006; Turner and Kumar, 2018; Tai et al., 2023). Each CesA subunit synthesizes a cellulose chain, and multiple subunits within the CSC collaborate to generate cellulose microfibrils. The CSC elongates cellulose chains by integrating glucose units from UDP-glucose through β-1,4-glycosidic linkages (Turner and Kumar, 2018; Pedersen et al., 2024).

The *Populus* genome contains 18 CesA genes (Suzuki et al., 2006). Compared with *Arabidopsis*, which has 10 CesA genes, *Populus* has almost twice as many CesA genes as *Arabidopsis*, with two homologous genes for each *Arabidopsis* CesA gene. Two types of CSCs have been identified in *Populus*. Type I CSCs include CesA4, CesA7A, CesA7B, CesA8A, and CesA8B, while Type II CSCs comprise CesA1A, CesA1B, CesA3C, CesA3D, CesA6E, and CesA6F. The two types of CSCs facilitate cellulose synthesis in wood xylem cells (Suzuki et al., 2006; Song et al., 2010). It is noteworthy that the two types of CSCs showed an effect on the formation of distinct structural properties of cellulose microfibrils in *Populus* wood (Xi et al., 2017; Abbas et al., 2020; Xu et al., 2021). In *Populus*, CesA4, CesA7A, CesA7B, CesA8A, and CesA8B may form a type of CSC that is associated with the deposition of highly crystalline cellulose (Song et al., 2010; Xi et al., 2017). Studies also showed that suppression of *PtrCesA7A* expression results in a significant reduction in cellulose crystallinity, whereas suppression of *PtrCesA3D* expression leads to an increase in cellulose crystallinity (Abbas et al., 2020). The temporal and spatial expression patterns of these *CesA* genes exhibit significant differences and may be related to their functional roles in cellulose deposition (Suzuki et al., 2006). These findings indicate the functional differentiation in the deposition of crystalline cellulose and the regulatory significance of *CesA*s in *Populus*, displaying their critical functions in wood cellulose deposition.

This study utilized a fiber cell-specific promoter to express *PdLTF1^AA^* for the repression of lignin biosynthesis while concurrently introducing cellulose synthase genes *(PdCesA4*, *PdCesA7A*, or *PdCesA8A*), which are highly expressed in xylem and may be related to the deposition of high crystalline cellulose to modify cellulose properties in xylem fiber cells. Results indicate that the biosynthesis of lignin and cellulose can be concurrently modified, resulting in changes to the wood fiber properties in *Populus*.

## 2 Materials and methods

### 2.1 Conditions for plant growth

This study utilized the hybrid *Populus* tree (*Populus deltoides × P. euramericana* cv. “Nanlin895”) (*Pd*), grown in a controlled-environment phytotron with the following parameters: a photoperiod of 14 hours of light and 10 hours of darkness, a relative humidity of 60%, and a constant temperature of 25°C.

### 2.2 Constructs and transformation

Based on a previous study (Gui et al., 2020), we constructed a vector named *PdDUF579-9p-35Smini-PdLTF1^AA^*. This vector utilizes the fiber cell-specific promoter *PdDUF579-9p* to drive the expression of *PdLTF1^AA^*, a mutant form of *PdLTF1* that can stably inhibit lignin synthesis. Meanwhile, the genes encoding *PdCesA4* (Potri.002G257900), *PdCesA7A* (Potri.006G181900), and *PdCesA8A* (Potri.011G069600) were cloned from Nanlin895. Using a fiber cell-specific promoter, *PdWND1B* (Potri.001G448400), the expression cassettes (*PdWND1Bp-35Smini-PdCesA4*, *PdWND1Bp-35Smini-PdCesA7A*, and *PdWND1Bp-35Smini-PdCesA8A*) were constructed for fiber cell-specific modification of cellulose in *Populus*. Following verification of the constructs’ accuracy through sequencing, they were introduced into *Populus* trees in accordance with the established protocol (Li et al., 2003). **Supplementary Table S1** lists the primers used for vector construction.

### 2.3 Quantitative RT-PCR

According to the previously described protocols (Li et al., 2025), xylem tissues were collected from the 15th internodes. Total RNA was extracted, and reverse transcription PCR (RT-PCR) was performed using the kit in accordance with the manufacturer’s protocol. The specific primers employed for each gene are provided in **Supplementary Table S1**.

### 2.4 Morphological analysis

Forty independent transgenic lines were generated. Among them, thirty-two independent transgenic lines were integrated with a single construct, five lines with two constructs, two lines with three constructs, and one line with four constructs were identified. The transgenic lines that had both *PdLTF1^AA^* and *CesA* cassettes were cloned using micro-cutting to generate multiple copies, which were then used as biological replicates. The transgenics grown in a phytotron were phenotypically assessed.

### 2.5 Microscopic observation of stem structure

Stem samples from the 15th internode were prepared for paraffin sectioning. In brief, stem samples were subjected to dehydration in a graded ethanol series, followed by clearing in xylene, and then immersed in paraffin. The stem tissues were sectioned at a thickness of 12 μm, stained with 1% toluidine blue for 20 minutes, and mounted with gum. The sections were then observed under an optical microscope. The thickness of the secondary cell wall (SCW) was examined via transmission electron microscopy. In brief, stem samples were sectioned transversely at 1 mm thickness and fixed under vacuum in 2.5% glutaraldehyde. They were then washed three times with 0.1 M phosphate buffered saline (PBS) buffer, fixed with 0.5% osmium tetroxide on a shaker, and washed again three times with 0.1 M PBS buffer. Subsequently, the samples were dehydrated in a graded ethanol series, embedded in epoxy resin, and polymerized at 50°C. Ultrathin sections were stained with lead citrate and uranyl acetate and then examined using a transmission electron microscope. The thickness of SCW was measured with ImageJ software.

### 2.6 Fiber length determination

The stem (the 15th internode) had its bark removed, was cut into 2-cm pieces, and then soaked overnight in a mixture of glacial acetic acid and 30% hydrogen peroxide at 60°C. After that, it was dyed with 1% safranine for 10 minutes and then photographed with a light microscope (Olympus, BX53). Next, the lengths of the fibers were measured using the Lorentzen & Wettre (LW) Fiber Tester from AB Lorentzen & Wettre in Kista, Sweden (Li et al., 2025).

### 2.7 Wood composition determination

Cellulose detection was performed as previously detailed (Gui et al., 2011). Xylem tissue from 16^th^-20^th^ internode cellulose content was measured as previously described (Foster et al., 2010b). Lignin detection was performed using phloroglucinol-HCl staining, following the described methods (Gui et al., 2011). Xylem tissue from 16^th^-20^th^ internode lignin content was measured as previously described (Foster et al., 2010a). Analytical thioacidolysis on alcohol insoluble residue (AIR) samples was performed as described previously (Yamamura et al., 2012). In brief, 5 mg of the AIR sample was treated with a reaction mixture composed of dioxane, ethanethiol, and boron trifluoride (35:4:1, v/v) to release lignin monomers. The lignin monomers were subsequently detected and quantified via gas chromatography-mass spectrometry (GC-MS). Tetracosane was used as an internal standard for quantification.

### 2.8 RNA sequencing

According to the previously described protocols (Li et al., 2025), total RNA was extracted from the xylem tissue of the 15th internode. The quality of the extracted RNA was verified, and mRNA was enriched using Oligo dT, fragmented, and then reverse transcribed into cDNA. After ligation with adaptors, the cDNA was sequenced on the NovaSeq X Plus platforms. Genes with an expression fold change of more than 2 were considered as differentially expressed genes (DEGs). Functional enrichment analysis and functional annotation were performed on the Meiji Cloud platform (https://analysis.majorbio.com/).

### 2.9 Statistical analysis

In this study, data were subjected to significance analysis using GraphPad Prism software, with one-way analysis of variance (ANOVA) utilized as the statistical method. Heat maps were visualized using TBtools.

## 3 Result

### 3.1 Morphological analysis of transgenic plants

**Figure 1:**
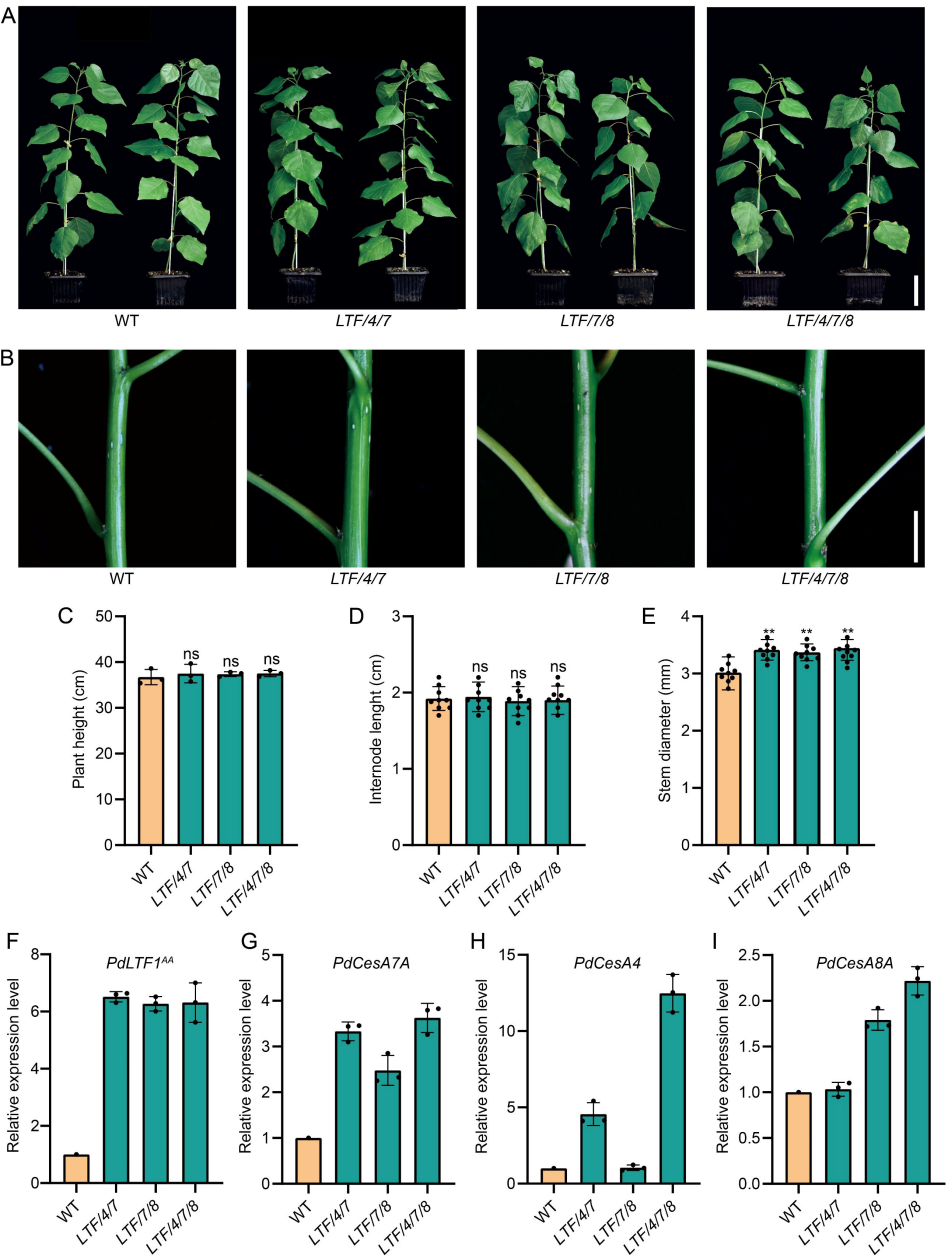
The growth morphologies of the fiber cell-specific regulation of *PdLTF1^AA^*, *PdCesA4*, *PdCesA7A*, and *PdCesA8A* in Populus. **(A, B)** Transgenics of *LTF/4/7, LTF/7/8, and LTF/4/7/8* at 2 months old. Scale bar, 6 cm in **(A)** and 0.6 cm in **(B)**. **(C)** Plant height, **(D)** Internode length (the 15^th^ internode), **(E)** Stem diameter (the 15^th^ internode). The *PdLTF1^AA^* **(F)**, *PdCesA7A* **(G)**, *PdCesA4* **(H)**, and *PdCesA8A* **(I)** expression in the transgenic xylem tissue compared to that in WT (the expression in WT is set to 1). The actin gene (Potri.001G309500) is used as a reference for expression normalization. The data are the mean values. The means ± standard deviation (SD) is calculated based on data obtained from three clonally propagated plants. Asterisks (**) indicate significant differences, P <0.01 (Student’s t-test). “ns” means no significant difference.

In earlier studies, we showed that the overexpression of *PdLTF1^AA^*, controlled by a fiber cell-specific promoter, effectively reduced lignin content without compromising normal plant growth (Gui et al., 2020). In this study, we investigate the specific expression of *PdLTF1^AA^* in fiber cells, in conjunction with *PdCesA4*, *PdCesA7A*, or *PdCesA8A* in *Populus*. We generated around 60 independent transgenic lines. After examination, the transgenic plants that had both *PdLTF1^AA^* and *CesA* genes, specifically the line called *LTF/4/7* with *PdLTF1^AA^*, *PdCesA4*, and *PdCesA7A*, the line named *LTF/7/8* with *PdLTF1^AA^*, *PdCesA7A*, and *PdCesA8A*, and the line labeled *LTF/4/7/8* with *PdLTF1^AA^*, *PdCesA4*, *PdCesA7A*, and *PdCesA8A*, were selected and cloned using micro-cutting. The propagated transgenics were grown in a phytotron for detail evaluation. In comparison to the wild type (WT), the transgenic plants *LTF/4/7*, *LTF/7/8*, and *LTF/4/7/8* exhibited comparable height growth but exhibited variations in stem diameter in a two-month period of growth (**Figure 1A, B**). The height and number of internodes showed no significant difference. However, the stem diameter increased by 13.7%, 12.2%, and 14.3% in *LTF/4/7*, *LTF/7/8*, and *LTF/4/7/8*, respectively (**Figure 1C-E**). In the transgenic plants, the integrated *PdLTF1^AA^*, *PdCesA4*, *PdCesA7A*, and *PdCesA8A* genes were expressed correctly in *LTF/4/7*, *LTF/7/8*, and *LTF/4/7/8*, respectively (**Figure 1F-I**).

### 3.2 Anatomical features of the transgenic stems

**Figure 2:**
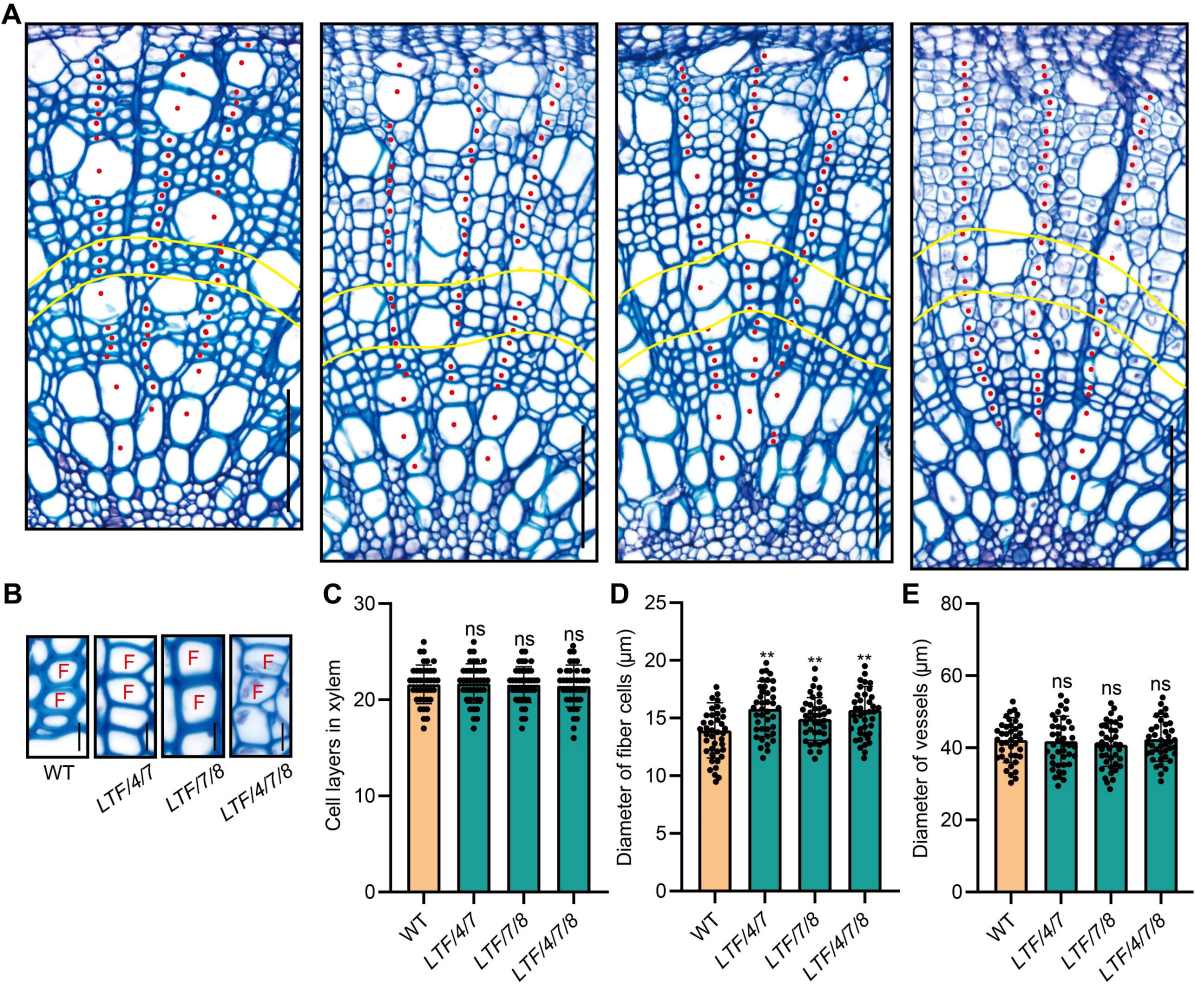
Stem cross anatomy of the *LTF/4/7*, *LTF/7/8*, and *LTF/4/7/8*. **(A)** Stem cross sections (the 15^th^ internode) of *LTF/4/7, LTF/7/8, and LTF/4/7/8* stained with toluidine blue. The red dots indicate xylem cells arranged in a file. The yellow curve marks an area of the xylem cells at the 12^th^ ~ 15^th^ layers in a file that was examined for cell morphology. Scale bar, 100 μm, **(B)** Cross view of xylem fibers. Scale bar, 13 μm. F, fiber cell. **(C)** Cell layers in xylem, **(D)** Diameter of fiber cells. **(E)** Diameter of vessels. The results are mean ± SD based on the determination of 40 cells from each of three clonally propagated plants. Asterisks (**) indicate significant differences, P <0.01 (Student’s t-test). “ns” means no significant difference.

**Figure 3:**
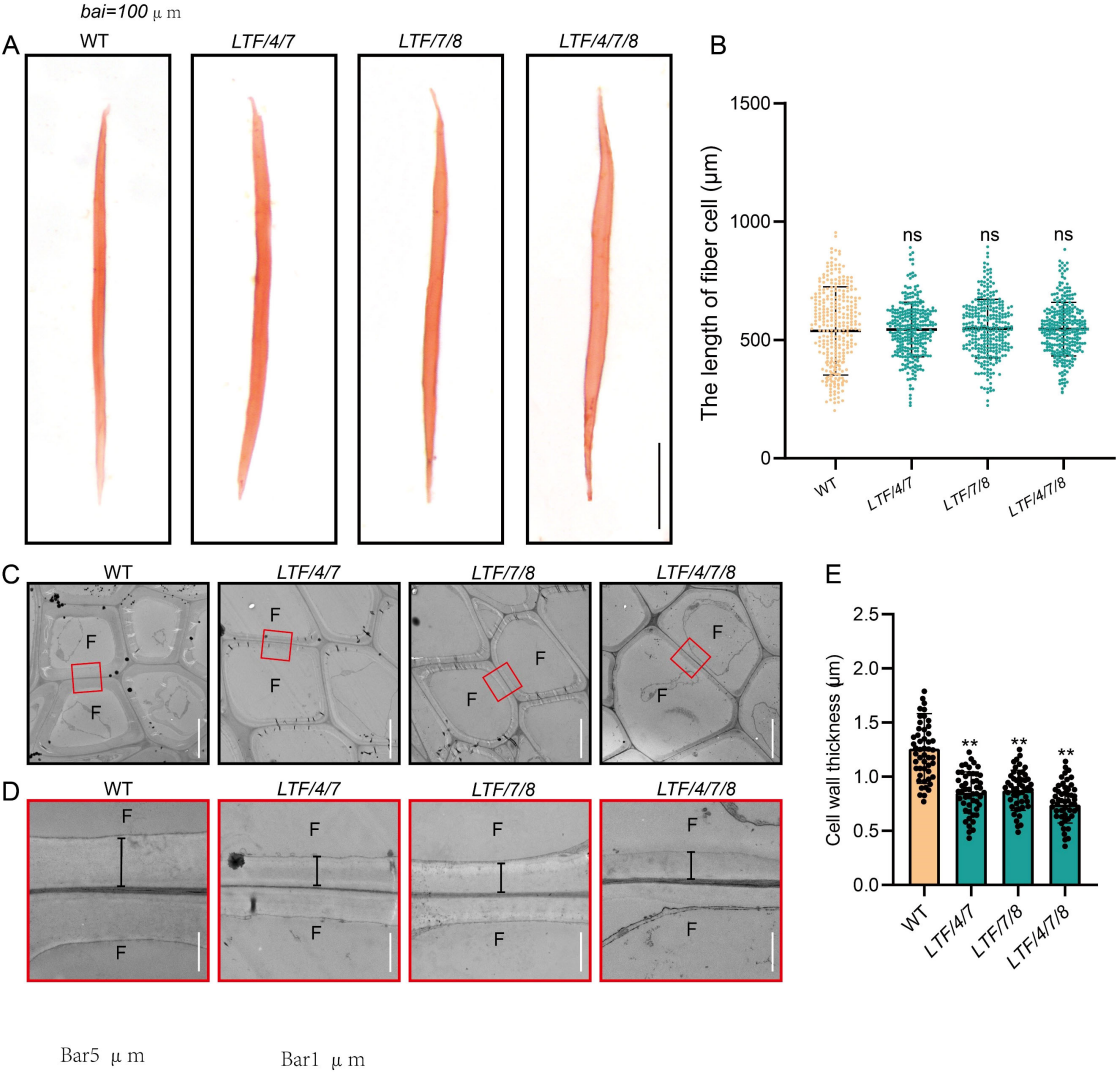
The 15^th^ internode of fiber cell length and cell wall thickness in *LTF/4/7, LTF/7/8, and LTF/4/7/8*. **(A)** Dissociated fibers from the 15^th^ internode were observed under an optical microscope. Scale bar, 100 μm. **(B)** The length of the dissociated fibers. **(C, D)** Transmission electron micrographs of the 15^th^ internode fiber cell wall in LTF/4/7, LTF/7/8, and LTF/4/7/8. F, fiber cell. Magnification of the fiber cell wall in marked red rectangles. The black lines indicate the thickness of the cell wall. Scale bar, 5 μm in **(C)** and 1 μm in **(D)**. **(E)** Cell wall thickness in *LTF/4/7*, *LTF/7/8*, and *LTF/4/7/8*. The length of fiber cells: mean ± SD, based on 200 fiber cells measured from the 15th internode of transgenics. Cell wall thickness: means ± SD of 50 fiber cells from the 15th internode of transgenics. Asterisks (**) indicate significant differences, P <0.01 (Student’s t-test). “ns” indicates no significant difference.

The altered stem diameter morphology observed in the transgenic plants suggests potential modifications in xylem development. To investigate this further, we examined the development of the stem internode. Results indicated that the transgenics displayed an increase in xylem width (**Figure 2A, B**). In contrast, the number of cell layers within the xylem tissue was similar to the WT (**Figure 2C**). Further examination of the size of cells in the xylem tissue showed that the diameter of fiber cells increased, but the diameter of vessel cells did not change much (**Figure 2D, E**). To further elucidate the characteristics of fiber cells in the transgenic lines, we performed longitudinal dissections of the stems. This analysis indicated that fiber cell length did not show a significant difference from that of the WT (**Figure 3A, B**). However, the transgenics *LTF/4/7*, *LTF/7/8*, and *LTF/4/7/8* exhibited a notable reduction in fiber cell wall thickness (**Figure 3C-E**). Overall, these results show that the transgenics *LTF/4/7*, *LTF/7/8*, and *LTF/4/7/8* have fiber cells that are wider but have thinner walls.

### 3.3 Cell wall composition in the transgenics

**Figure 4:**
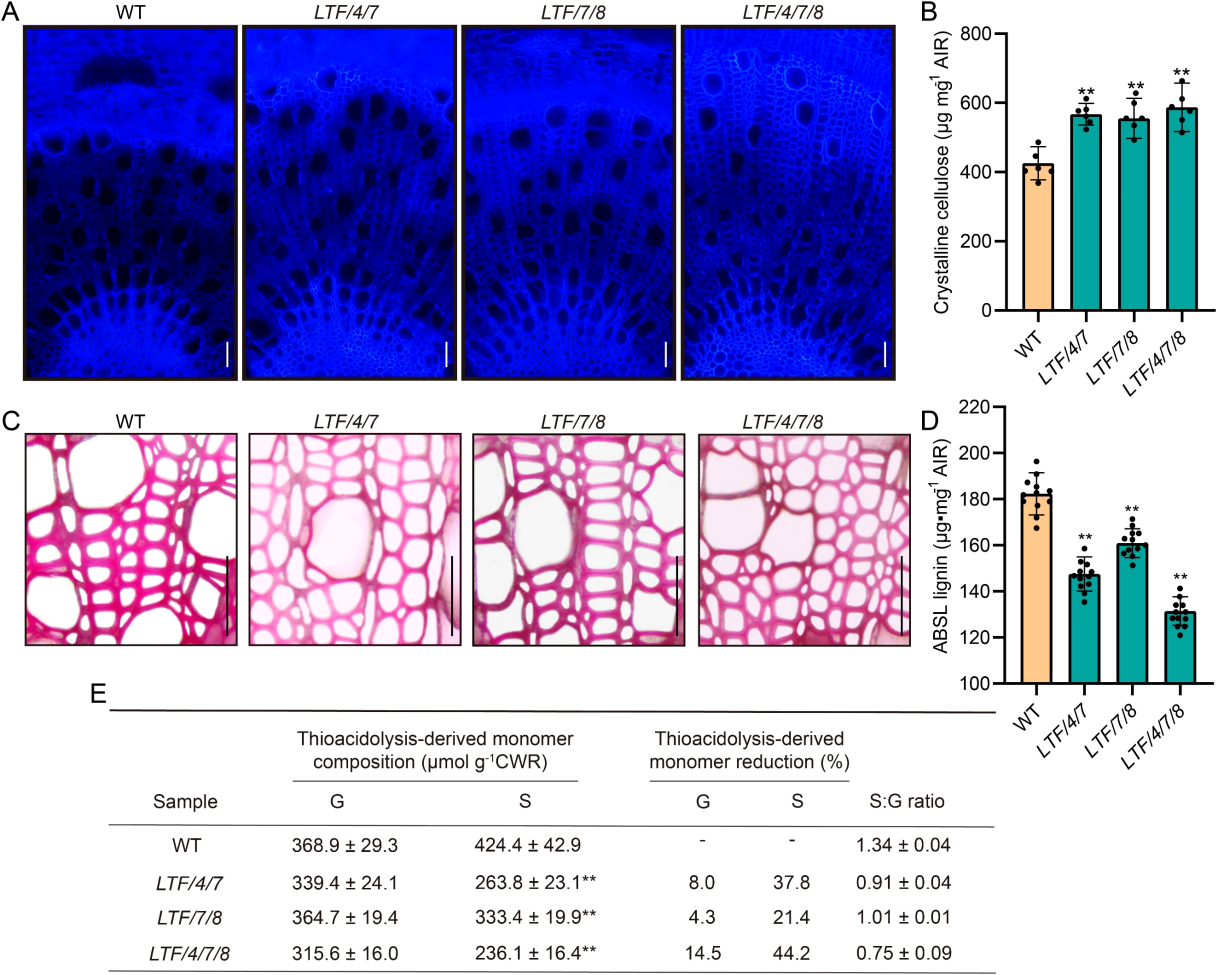
The cell wall composition was measured in the 16^th^ to 20^th^ internode of LTF/4/7, LTF/7/8, and LTF/4/7/8. **(A)** The cross-sectional stems of LTF/4/7, LTF/7/8, and LTF/4/7/8 were stained with cellulose dyes. Bar, 100μm. **(B)** Crystalline cellulose. **(C)** The cross-sectional stems of LTF/4/7, LTF/7/8, and LTF/4/7/8 were stained with phloroglucinol. Bar, 100μm. **(D)** Lignin content. **(E)** Lignin monomers. Cellulose content: means ± SD of 6 samples from three clonally propagated plants. Lignin content: means ± SD of 12 samples. Lignin monomers: means ± SD of 3 samples. Asterisks (**) indicate significant differences (P < 0.01, Student’s t-test).

To investigate the composition of fiber cell walls in transgenic plants, cellulose staining demonstrated that the 14^th^ internode of these transgenic lines exhibited significantly stronger staining intensity compared to that of the WT (**Figure 4A**). Quantitative analysis of crystalline cellulose content corroborated these results, revealing increases of 33.3%, 30.6%, and 38.1% in *LTF/4/7*, *LTF/7/8*, and *LTF/4/7/8*, respectively (**Figure 4B**). Phloroglucinol staining indicated a significant reduction in staining intensity in the transgenics compared to the WT, suggesting a decrease in lignin content (**Figure 4C**). Lignin content analysis was consistent with the phloroglucinol staining results, showing reductions of 19.1%, 11.7%, and 27.9% in *LTF/4/7A*, *LTF/7/8*, and *LTF/4/7/8*, respectively (**Figure 4D**). Additionally, quantitative analysis of lignin monomers in the transgenic plants revealed a decrease in both G-type and S-type lignin monomer contents. Notably, the decrease in S-type lignin monomers was significantly more pronounced than that of G-type monomers, leading to a substantial reduction in the S/G ratio (**Figure 4E**). Among the transgenic lines, *LTF/4/7/8* exhibited the most significant reduction in S-type lignin monomer content, with a decrease of 44.2% (**Figure 4E**). These findings indicate that the overexpression of *PdLTF1^AA^*, *PdCesA4*, *PdCesA7A*, and *PdCesA8A* modified xylem fiber cell wall biosynthesis, thereby leading to an increase in fiber cell diameter.

### 3.4 The transcriptional landscape in the developing xylem of the transgenic plants was altered

**Figure 5:**
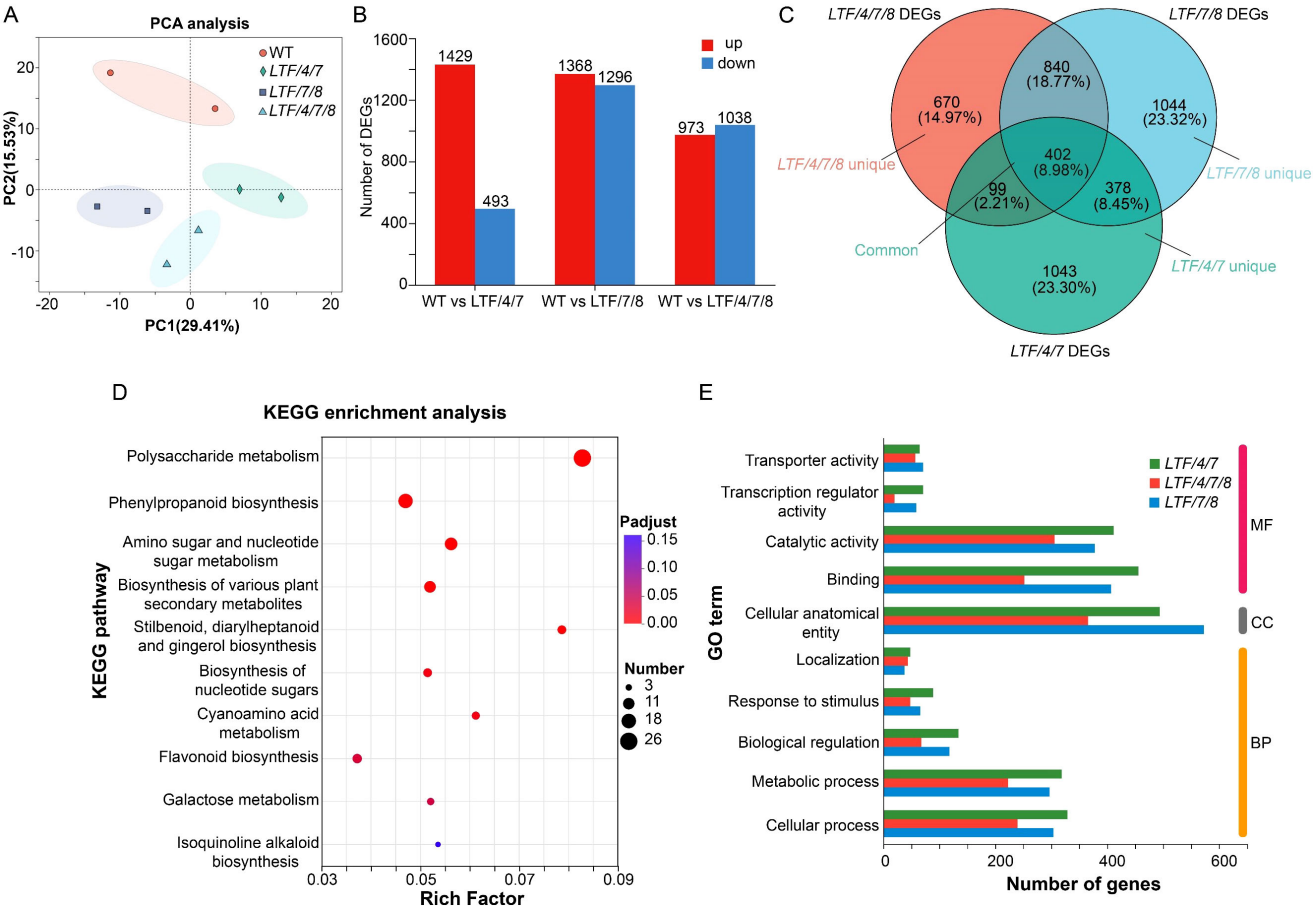
RNA sequencing of the stem xylem tissue in *LTF/4/7, LTF/7/8, LTF/4/7/8*, and WT. **(A)** PCA analysis of WT, *LTF/4/7*, *LTF/7/8*, and *LTF/4/7/8* samples. The horizontal axis represents the contribution degree of principal component 1 (PC1) in the two-dimensional graph to the distinguished samples, and the vertical axis represents the contribution degree of principal component 2 (PC2) in the two-dimensional graph to the distinguished samples. **(B)** DEGs were identified between *LTF/4/7* and WT, *LTF/7/8* and WT, and *LTF/4/7/8* and WT. **(C)** The Venn diagram analysis of DEGs in *LTF/4/7*, *LTF/7/8*, and *LTF/4/7/8.* **(D)** KEGG (Kyoto Encyclopedia of Genes and Genomes) enrichment analysis was performed on the common DEGs in *LTF/4/7*, *LTF/7/8*, and *LTF/4/7/8*. p-adjust and rich factor are indicated. **(E)** GO annotation was performed on the unique DEGs in *LTF/4/7*, *LTF/7/8*, and *LTF/4/7/8*. MF, molecular function. CC, cellular component. BP, biological process.

**Figure 6:**
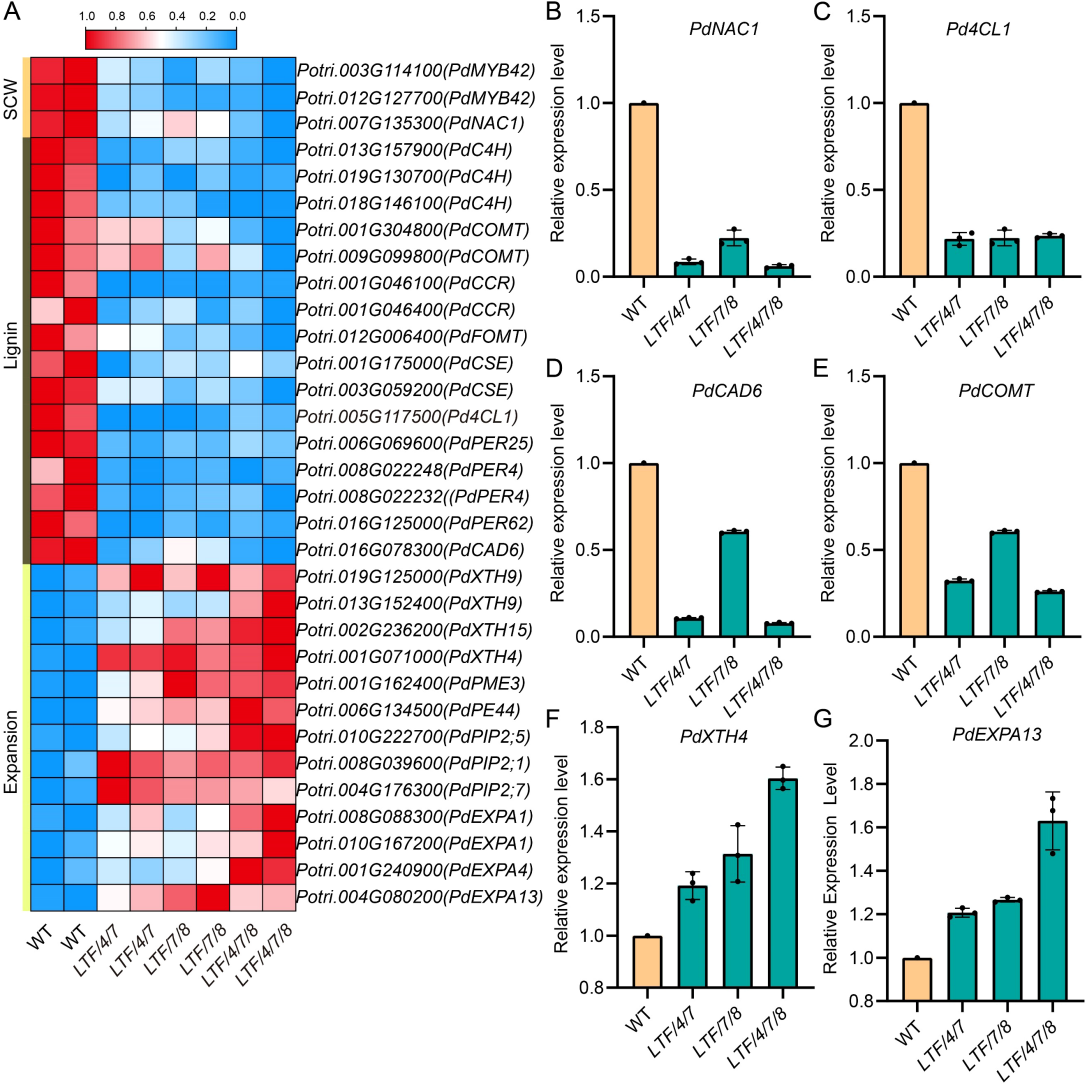
The common DEGs were enriched in cell wall organization and lignin biosynthesis. **(A)** Heat maps of the genes associated with cell wall organization and lignin biosynthesis. **(B)** RT-PCR validation of the differential expression of key genes involved in secondary cell wall biosynthesis (*PdNAC1*), lignin biosynthesis (*Pd4CL1, PdCAD6, PdCOMT*), expansion (*PdXTH4, PdEXPA13*). Data are means ± SD of 3 independent plants.

Given the observed modifications in fiber cell dimension in the transgenics, we conducted transcriptomic analysis to elucidate how transcriptional activities were regulated by integration of *PdLTF1^AA^*, *PdCesA4*, *PdCesA7A*, and *PdCesA8A* genes. Developing xylem tissue was harvested for RNA sequencing analysis (**Figure 5A**). RNA sequencing analysis identified a substantial number of differential expressed genes (DEGs), which had a fold change > 2 in the transgenic plants compared with the WT. Specifically, the *LTF/4/7* exhibited 1,922 DEGs, including 1,429 genes that were significantly upregulated and 493 genes that were significantly downregulated. The *LTF/7/8* exhibited a total of 2,664 DEGs, consisting of 1,368 significantly upregulated genes and 1,296 significantly downregulated genes. The *LTF/4/7/8* exhibited 2,011 DEGs, comprising 973 genes that were considerably upregulated and 1,038 genes that were significantly downregulated (**Figure 5B**, **Supplementary Table S2**). Through the examination of DEGs in *LTF/4/7*, *LTF/7/8*, and *LTF/4/7/8* via a Venn diagram, we identified that 402 DEGs were shared among these transgenic plants (**Figure 5C**). The common DEGs predominantly participate in the metabolism of polysaccharides, as well as the biosynthesis of phenylpropanoids and associated secondary metabolites (**Figure 5D**). Conversely, it is noteworthy that the distinct DEGs found in the three types of transgenic plants were associated with various biological activities related to cell wall formation in an analogous distribution (**Figure 5E**). Among the common DEGs, those related to SCW deposition, such as PdNAC1, and lignin biosynthesis, including *Pd4CL1, PdCAD6*, and *PdCOMT*, were significantly downregulated (**Figures 6A–E**). This downregulation is consistent with the observed changes in the characteristics of the fiber cell wall. Moreover, the expression of genes associated with cell wall expansion, such as *PdXTH4* and *PdEXPA13*, was significantly upregulated (**Figures 6F–G**). This upregulation correlates with the observed increase in fiber cell diameter in the transgenic plants. The data demonstrate that modification of lignin biosynthesis, in conjunction with regulation of various CesA genes in fiber cells, led to changes in the transcriptional activity associated with cellulose and lignin deposition. Because the transgenic plants did not fully produce all the *CesA* combinations, it is important to further investigate how the different *CesA*s control cellulose deposition.

## 4 Discussion

Wood, a natural and renewable resource, is widely used in various fields, including pulping and papermaking, construction and furniture manufacturing, and energy and biomass uses. Wood comprises many cell types, with fiber cells constituting the primary structural component. The chemical composition of fiber cell walls directly affects the characteristics of wood. Thus, genetic engineering of fiber cell walls is regarded as an effective approach to improve wood characteristics (Chanoca et al., 2019; Zhu and Li, 2024).

The amounts of lignin and cellulose, as primary constituents of wood cell walls, significantly influence the qualities and applications of wood. Therefore, regulating lignin and cellulose synthesis to generate wood with appropriate cell wall components is essential for enhancing wood application value. Many studies have been undertaken in the past thirty years on the regulation of lignin biosynthesis and the genetic modification of lignin composition and structure (Boerjan et al., 2003; Chanoca et al., 2019; Li et al., 2024; Zhu and Li, 2024). The regulation of lignin deposition often influences field growth (Voelker et al., 2010; Zhu and Li, 2024). Such an effect may be due to the utilization of constitutive promoters to regulate lignin deposition, potentially altering the lignin composition in all cells, including vessel cell walls, which are crucial for the long-distance transport of water within the vessel. Recent studies indicate that controlling lignin deposition specifically in fiber cells has little effect on growth (Cao et al., 2020; De Meester et al., 2020; Gui et al., 2020). Altering lignin production in the fiber cell wall did not affect the lignin composition in the vessel cell wall, meaning it did not impact long-distance water transport and therefore did not affect growth in field conditions (Cao et al., 2020; Gui et al., 2020).

Meanwhile, studies have also been conducted on modifying cellulose biosynthesis in trees (Coleman et al., 2009; Maloney and Mansfield, 2010; Joshi et al., 2011; Yu et al., 2014; Nayeri et al., 2022). Our previous studies identified two types of CSCs participating in wood formation, with distinct gene influences on crystalline cellulose synthesis (Song et al., 2010; Xi et al., 2017). One type of CSA was linked to the deposition of crystalline cellulose, while the other may be linked to the biosynthesis of cellulose with a low degree of crystallinity. Evidence indicates that the regulation of cellulose synthase genes influences both cellulose content and cellulose crystalline properties (Yu et al., 2014; Xi et al., 2017; Abbas et al., 2020; Pedersen et al., 2024). In this study, we enhanced the expression of cellulose synthase genes that are related to a high degree of crystalline cellulose in fiber cells while inhibiting lignin biosynthesis. The transgenics showed that the lignin content decreased and the cellulose content increased, especially the increase of crystalline cellulose. The results of this study further suggest that cellulose with different crystalline structures may be related to different *CesA* genes; still, this conclusion warrants further corroboration through more comprehensive studies.

While lignin and cellulose can be individually modified, there have been no previous studies published on the concurrent regulation of lignin and cellulose biosynthesis. The results of the current study indicated modifications in both cell wall constituents, characterized by a reduction in lignin and an increase in cellulose. The study revealed the possibility of inhibiting lignin biosynthesis metabolism while simultaneously enhancing cellulose synthesis. The biosynthesis of cell wall components involves the coordinative function of multiple levels of gene networks, encompassing transcription factors, hormone signaling, cell wall biosynthesis enzyme genes, etc (Zhong et al., 2019; Zhang et al., 2020; Zhu and Li, 2024). It appears that both suppressing the lignin biosynthesis pathway and enhancing the cellulose synthesis process can happen at the same time; thus, manipulating several processes that produce cell walls at the same time could be crucial, enabling the development of various strategies to bioengineer trees with well-designed cell walls.

## 5 Conclusion

Lignin and cellulose are the primary components of wood cell walls, significantly influencing the characteristics and uses of wood. Many studies have indicated that the lignin and cellulose content of wood can be altered using genetic engineering; nevertheless, it remains to be established if lignin and cellulose can be concurrently modified to provide wood raw materials with desirable chemical composition. This study demonstrated how the deposition of cellulose and lignin in fiber cells, which are the main cells in wood, can be controlled, allowing both of these components in wood to be modified at the same time. Transgenic plants exhibited an enlarged diameter of wood fiber cells, accompanied by transcriptional changes in genes related to cell wall remodeling and polysaccharide synthesis during xylem development. These findings enable the development of an innovative technical strategy for engineering chemical composition in wood and for the bioengineering of trees with designated wood characteristics.

## Data Availability

The accession number for our deposited data is CRA028446. The data is publicly accessible at the following link: https://ngdc.cncb.ac.cn/gsa.
